# Use of hand hygiene agents as a surrogate marker of compliance in Hungarian long-term care facilities: first nationwide survey

**DOI:** 10.1186/s13756-015-0069-0

**Published:** 2015-07-31

**Authors:** Rita Szabó, Júlia Morvai, Fernando Bellissimo-Rodrigues, Didier Pittet

**Affiliations:** National Center for Epidemiology, Department of Hospital Epidemiology and Hygiene, Albert Flórián út 2-6, Budapest, 1097 Hungary; Semmelweis University, School of PH.D. Studies, Vas utca 17, Budapest, 1088 Hungary; Infection Control Programme, University of Geneva Hospitals and Faculty of Medicine, World Health Organization (WHO) Collaborating Centre on Patient Safety, Rue Gabrielle-Perret-Gentil 4, Geneva, 1205 Switzerland

**Keywords:** Hand hygiene, Alcohol-based handrub, Compliance, Long-term care facilities, Hungary

## Abstract

**Background:**

Hand hygiene practice is an important measure for preventing infections in long-term care facilities (LTCFs). However, low compliance with hand hygiene has been reported in a number of studies. The purpose of this study was to provide an overview of the first reference data collected on alcohol-based handrub (ABHR) and antiseptic soap consumption, as surrogate markers for hand hygiene compliance by healthcare workers (HCWs) in Hungarian LTCFs. The objective was to inform stakeholders on the need of hand hygiene improvement in these settings.

**Methods:**

Between 5 May and 30 September 2014, we conducted a nationwide, cross-sectional survey using a standardized self-administered questionnaire; all Hungarian LTCFs were eligible. The Statistical Package for Social Sciences (SPSS) version 20.0 was used for data analysis.

**Results:**

The questionnaire was completed by 354 LTCFs, representing 24 % of all Hungarian LTCFs. In total, the median consumption of ABHR and antimicrobial soap was 15.5 L (IQR, 0–800 L) and 60 L (IQR, 0–1,680 L) per LTCFs, and 2.2 mL (IQR, 0.4–9.1 mL) and 12.1 mL (IQR, 0.7–32.8 mL) per HCWs in 2013, respectively. The estimated number of hand hygiene actions was 0.6 hygienic handrub/HCW per day (IQR, 0–12.8/HCWs) and 2.4 hygienic handwashing/HCW per day (IQR, 0–21.9/HCWs; *P* = .001), respectively.

**Conclusions:**

This study suggests that non-compliance with hand hygiene is a significant problem in Hungarian LTCFs. Based on our results, there is an urgent need for a nationwide multimodal hand hygiene promotion strategy including education and performance monitoring and feedback in all LTCFs. Furthermore, monitoring of ABHR consumption constitute an additional component of the existing National Nosocomial Surveillance system.

## Background

Healthcare-associated infections (HAIs) are frequent among residents of long-term care facilities (LTCFs) over the world. Recent reports indicated that the prevalence of HAIs in these settings is 2.1 % in Hungary and up to 8.7 % in other European countries [1–7]. The burden of HAIs is important and includes high morbidity and mortality, and increased treatment costs [8]. HAIs are caused by pathogens directly transmitted by healthcare workers’ (HCWs) hands or from the external environment to the susceptible resident [9–12].

Since the famous Hungarian physician Ignaz Semmelweis’s observation in 1847, hand hygiene is considered the simplest and most important measure to reduce causative pathogens cross-transmission and prevent HAIs both in acute healthcare settings and LTCFs [13, 14]. Despite relevant evidences and the simplicity of this procedure, compliance with recommended hand hygiene practices is extremely low in LTCFs, between 4 and 25.7 % [12, 15–17]. Main reasons for non-compliance include inadequate knowledge of guidelines for hand hygiene, insufficient time for hand hygiene practice, skin irritation, and the inconvenient location of dispensers [18].

Facing these barriers, the World Health Organization (WHO) published hand hygiene guidelines for outpatient, home-based care and LTCFs in 2012, recommending the use of alcohol-based handrub (ABHR) as the preferred hand hygiene agent, preferably immediately accessible at the point of patient care (i.e., where care is provided) [19]. ABHR has a broad antimicrobial spectrum, short action time (20–30 s), good skin tolerability and can be made easily accessible, including in the form of pocket-sized containers. In addition, recording the consumption of ABHR can constitute a rapid and inexpensive surrogate measure (i.e. indirect method) of hand hygiene compliance [18, 19].

Despite the relative simplicity of indirect methods to monitor hand hygiene activities, no such research has been conducted in LTCFs worldwide. Therefore the main objective of our study was to provide an overview of the first baseline data collected on ABHR and also antimicrobial soap consumption as surrogate parameters for hand hygiene compliance in Hungarian LTCFs.

## Methods

We conducted a nationwide survey between 5 May 2014 and 30 September 2014. Invitation letters explaining the objective of the study were sent to all Hungarian LTCFs (*n* = 1485), including general nursing homes, psychiatric LTCFs, LTCFs for the physically disabled people, rehabilitation centres and other LTCFs (e.g., palliative care centres, mixed LTCFs), i.e. all LTCFs registered in the Hungarian Rehabilitation and Social Institute. Follow-up letters for non-respondents were mailed monthly during the study period (June 2, July 2, August 4 and September 1, 2014). Ethical approval was received from the directors of participating LTCFs and all the participants signed the informed consent. Participants were guaranteed of the privacy and anonymity of the information provided.

A self-developed, 14-items questionnaire was used as a tool to collect information retrospectively for the year 2013. The structured questionnaire included four sessions: 1) demographic characteristics (e.g., professional category of directors, type of LTCF, total number of residents, total number of HCWs); 2) consumption of ABHR (e.g., consumption in litres, location of ABHR dispensers, number of pocket-sized containers); 3) consumption of antimicrobial soap (e.g., consumption in litres, location of antimicrobial soap dispensers); and 4) education (e.g., type of hand hygiene training, location of reminder materials).

Daily consumption of ABHR per HCWs was calculated by dividing the total amount of ABHR consumption (mL/year) by the total number of HCWs divided by 365. The estimated number of hygienic handrub action per HCWs was calculated by dividing the total amount of daily ABHR consumption (mL/HCWs) by 3 (3 ml is the recommended amount of ABHR for one hygienic handrub action). Daily consumption of antimicrobial soap per HCWs was calculated by dividing the total amount of consumption of antimicrobial soap (mL/year) by the total number of HCWs divided by 365. The estimated number of hygienic handwashing per HCWs was calculated by dividing the total amount of daily consumption of antimicrobial soap (mL/HCWs) by 5 (5 ml is the recommended amount of antimicrobial soap for one hygienic handwashing action).

Descriptive statistics were used as appropriate. Categorical variables were described as number (%), while continuous variables were reported as number (%) and median (interquartile range, IQR). The Mann-Whitney *U* test was used to compare the differences between the number of hygienic handrub and hygienic handwashing actions stratified by type of LTCFs. A *P* value of less than 0.05 was considered statistically significant for all analysis. The Statistical Package for Social Sciences (SPSS, SAS Institute Inc, Cary, NC, USA) version 20.0 was used for analysis of the data.

## Results

### Demography

In total, completed questionnaires were obtained from 354 LTCFs, for an overall response rate of 24 %. Responses were distributed among all specialities, with the highest number of responses from general nursing homes (*n* = 278, 78.5 %), followed by LTCFs for the physically disabled (*n* = 40, 11.3 %), psychiatric LTCFs (*n* = 19, 5.4 %), other LTCFs (*n* = 13, 3.7 %) and rehabilitation centres (*n* = 4, 1.1 %). The majority of the LTCF directors were nurses (*n* = 181, 51.1 %), followed by social workers (*n* = 106, 30 %), doctors (*n* = 5, 1.4 %), and others (*n* = 62, 17.5 %) including priests and dieticians. The median number of residents and HCWs was 43 (IQR, 9–251) and 40.7 (IQR, 5–320).

### Availability of hand hygiene agents

**Fig. 1:**
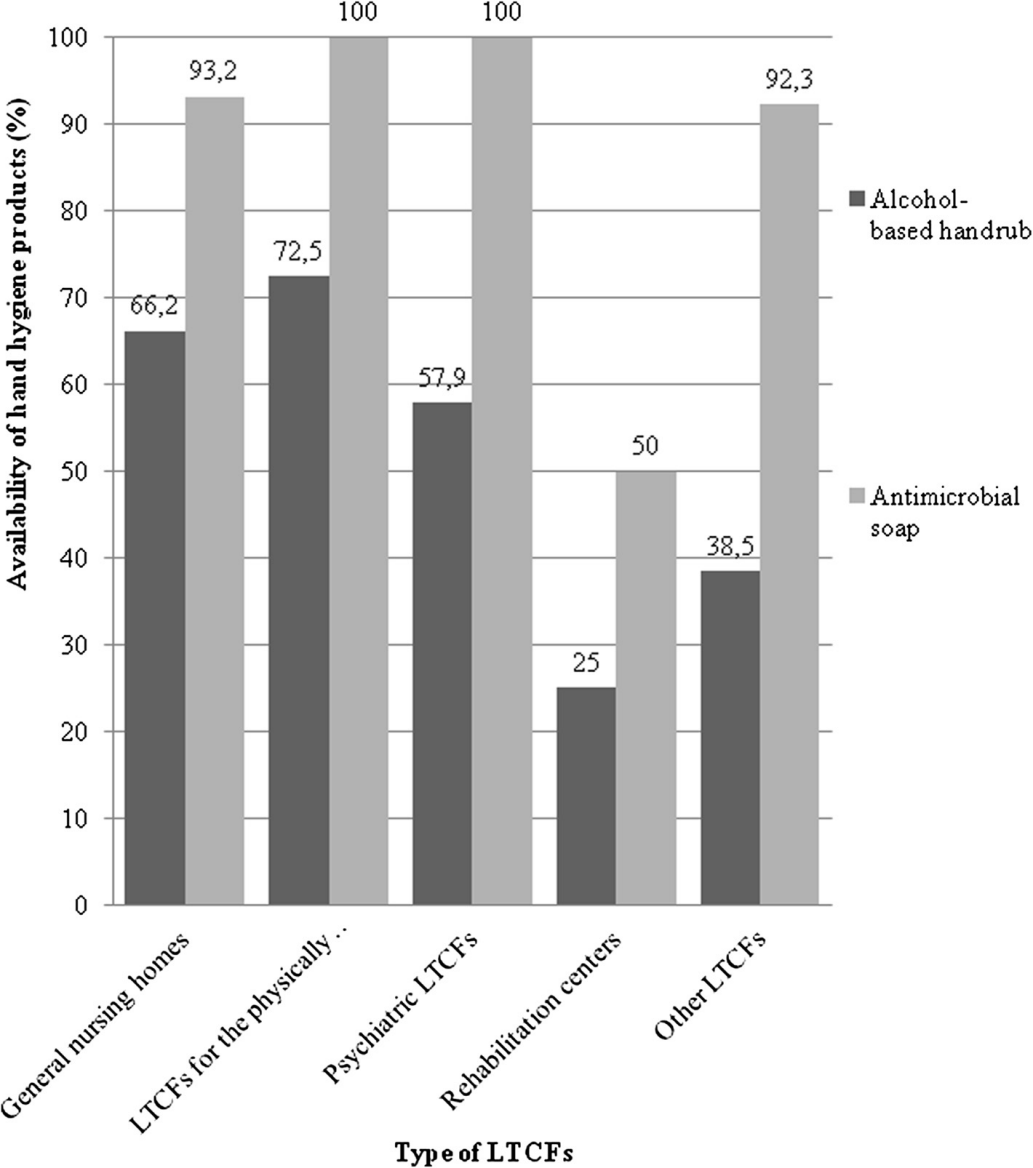
Availability of alcohol-based handrub and antimicrobial soap stratified by types of long-term care facilities (LTCFs), Hungary, 2013

Most LTCFs had ABHR (*n* = 230, 64.9 %) and antimicrobial soap (*n* = 332, 93.8 %) available in 2013. The availability of ABHR was the most common in LTCFs for the physically disabled (*n* = 29, 72.5 %) and the less frequent in the rehabilitation centres (*n* = 1, 25 %). The availability of antimicrobial soap was the most common in the LTCFs for the physically disabled (*n* = 40, 100 %) and psychiatric LTCFs (*n* = 19, 100 %), while it was the less frequent in the rehabilitation centres (*n* = 2, 50 %). The distribution of ABHR and antimicrobial soap availability stratified by types of LTCFs is presented in Fig. 1.

### Consumption of hand hygiene agents

In total, the median annual consumption of ABHR and antimicrobial soap was 15.5 L (IQR, 0–800 L) and 60 L (IQR, 0–1,680 L) per LTCFs, and 2.2 mL (IQR, 0.4–9.1 mL) and 12.1 mL (IQR, 0.7–32.8 mL) per HCWs in 2013, respectively. At the institutional level, ABHR and antimicrobial soap consumption was the highest in the rehabilitation centre (800 L) and in the psychiatric LTCFs (120 L; IQR, 12–300 L), while the less frequent in the general nursing homes (15 L; IQR, 0–415 L) and in the rehabilitation centres (21.5 L). At the HCWs’ level, ABHR and antimicrobial soap consumption was the highest in the other LTCFs (3.5 mL/HCWs; IQR, 0.8–4.7 mL/HCWs vs. 16.8 mL/HCWs; IQR, 0.3–59.5 mL/HCWs), while the less frequent in the LTCFs for the physically disabled people (1.5 mL/HCWs; IQR, 0–4.4 mL/HCWs) and in the rehabilitation centres (0.1 mL/HCWs). Hand hygiene agents consumption stratified by types of LTCFs is presented in Tables 1 and 2.

**Table 1:** Baseline data on alcohol-based handrub (ABHR) consumption among 230 long-term care facilities (LTCFs) stratified by types of settings, Hungary, 2013

Type of LTCF	Number of LTCFs *n* (%)	Number of healthcare workers (HCWs)	Annual consumption of ABHR in litres	Distribution of ABHR consumption, mL/HCWs per day		
		Median (IQR)	Median (IQR)	10^th^ percentile	Median	90^th^ percentile
General nursing homes	184 (80)	19.5 (4–150)	15.0 (0–415)	0.4	2.2	9.4
LTCFs for the physically disabled people	29 (13)	54.0 (6–136)	24.0 (0–120)	0	1.5	4.4
Psychiatric LTCFs	11 (4.8)	52.0 (23–310)	45.0 (10–565)	0.4	2.5	5.7
Rehabilitation centres	1 (0.4)	75.0 (–)	800 (–)	–	–	–
Other LTCFs	5 (2.2)	12.0 (5–30)	20.0 (6–33)	0. 8	3.5	4.7
Total	230	23.0 (4–310)	15.5 (0–800)	0.4	2.2	9.1

**Table 2:** Baseline data on antimicrobial soap consumption among 332 long-term care facilities (LTCFs) stratified by types of settings, Hungary, 2013

Type of LTCF	Number of LTCFs *n* (%)	Number of healthcare workers (HCWs)	Annual consumption of antimicrobial soap in litres	Distribution of antimicrobial soap consumption, mL/HCWs per day		
		Median (IQR)	Median (IQR)	10^th^ percentile	Median	90^th^ percentile
General nursing homes	259 (78)	18.0 (22–166)	50.0 (0–1200)	0.6	12.1	32.6
LTCFs for the physically disabled people	40 (12)	51.0 (5–136)	77.5 (0–1680)	2.2	9.8	26.3
Psychiatric LTCFs	19 (5.7)	40.0 (12–310)	120.0 (12–300)	1.4	13.9	39.1
Rehabilitation centres	2 (0.6)	23.5 (5–42)	21.5 (0–43)	0	0.1	–
Other LTCFs	12 (3.6)	9.0 (5–30)	42.0 (5–200)	0.3	16.8	59.5
Total	332	20.0 (22–310)	60 (0–1680)	0.7	12.1	32.8

### Hand hygiene compliance

The estimated number of hand hygiene actions varied by types of LTCFs (Table 3). Overall, the median number of daily hygienic handrub and hygienic handwashing action in Hungarian LTCFs was 0.6/HCWs per day (IQR, 0–12.8/HCWs) and 2.4/HCWs per day (IQR, 0–21.9/HCWs; *P* = .001), respectively. The estimated number of hygienic handrub and hygienic handwashing actions were the highest in rehabilitation centres (9.7/HCWs per day) and in other LTCFs (3.3/HCWs per day; IQR, 0–12.5/HCWs per day), while smallest estimated were calculated from LTCFs for the physically disabled people (0.5/HCWs per day; IQR, 0–2.5/HCWs per day) and in the only rehabilitation centre (0.0/HCWs per day; IQR, 0–0.9/HCWs per day).

**Table 3:** Estimated number of hand hygiene actions per HCW per day in long-term care facilities (LTCFs) stratified by types of settings, Hungary, 2013

Type of LTCF	Hand hygiene actions (N) per HCW per day	
	Hygienic handrub	Hygienic handwashing
	Median (IQR)	Median (IQR)
General nursing homes	0.7 (0–12.8)	2.4 (0–21.9)
LTCFs for the physically disabled people	0.5 (0–2.5)	1.9 (0–17.4)
Psychiatric LTCFs	0.9 (0–1.9)	2.8 (0–7.9)
Rehabilitation centres	9.7 (–)	0.0 (0–0.9)
Other LTCFs	1.0 (0–3.7)	3.3 (0–12.5)
Total	0.7 (0–12.8)	2.4 (0–21.9)

### Accessibility of hand hygiene agents

Both type of hand hygiene agents were the most accessible in the nursing rooms (*n* = 210, 91.3 % and *n* = 282, 84.9 %, respectively). In addition, ABHR was the most available in the doctor’s rooms (*n* = 170, 73.9 %) and in the examination rooms (*n* = 126, 54.8 %), while the antimicrobial soap was the most accessible in the toilet for HCWs (*n* = 275, 82.3 %) and in the examination rooms (*n* = 242, 72.9 %).

### Pocket-sized ABHR containers

83.9 % of participating LTCFs (*n* = 193) provided pocket-sized ABHR containers for their HCWs in 2013, especially in the general nursing homes (*n* = 157, 81.3 %) followed by LTCFs for the physically disabled people (*n* = 21, 10.9 %) and psychiatric LTCFs (*n* = 11, 5.7 %). Despite of the fact that rehabilitation centre used the most ABHR, pocket-sized containers were not available in these LTCFs.

### Hand hygiene trainings

A large majority of the participating directors (*n* = 333, 94.1 %) reported to have organized hand hygiene training for care professionals in the previous year. However, only 10.8 % of LTCFs (*n* = 36) proposed a complete hand hygiene training including theoretical and practical sessions. In 43.8 % of LTCFs (*n* = 146), hand hygiene trainings were exclusively provided by the display of posters. Poster reminders were the most available in the nursing rooms (*n* = 261, 73.7 %), followed by the doctors’ rooms (*n* = 196, 55.4 %) and LTCFs kitchen (*n* = 153, 43.2 %).

## Discussion

While several studies have measured HCWs hand hygiene compliance by direct observation in LTCFs, very few have estimated the number of hand hygiene actions by indirect methods [18–20]. Monitoring the consumption of ABHR and antimicrobial soap—as surrogate measures—is informative, simple, inexpensive, easily feasible in all healthcare sectors and at a large scale, consumes few resources, and has a reasonable to good correlation with observed compliance levels [21–25]. Therefore, we estimated hand hygiene practices of HCWs in Hungarian LTCFs and provided an overview to inform policy decision makers on the need for hand hygiene improvement in such settings.

Based on the results of the ‘Healthcare-associated infections and Antimicrobial use in European Long-Term care facilities’ (HALT-2) study, ABHR and antimicrobial soap were available in 90.7 % (*n* = 894) and 98.2 % (*n* = 968) of European LTCFs, while 67.8 % (*n* = 61) and 98.9 % (*n* = 89) of participating Hungarian LTCFs reported using ABHR and antimicrobial soap, respectively [26]. The results of the current study are in keeping with the Hungarian findings of HALT-2 study: 64.9 % (*n* = 230) and 93.8 % (*n* = 332) of participating Hungarian LTCFs provided ABHR and antimicrobial soap, respectively, in 2013. In the European-wide HALT-2 study, 836 LTCFs reported consumption data of ABHR in litres used in 2012. A median ABHR use of 73.5 litres per year was reported, ranging from 0 to 9165 liters [26]. The current Hungarian results are lower than the European findings in 2012: the median ABHR consumption was 40.5 litres (IQR, 0-800 litres) in the previous year. In regard to the antimicrobial soap consumption data, especially from LTCFs, there is no related data in the literature. In Hungarian LTCFs, the median estimated number of hand hygiene action is very low: 0.6/HCWs per day for hygienic handrub and 2.4/HCWs per day for hygienic handwashing, respectively. It was particularly low in LTCFs for the physically disabled people (0.5/HCWs) and in the only rehabilitation centre (0.0/HCWs). However, both types of hand hygiene agents as well as poster reminders were widely available, and most LTCFs provided pocket-sized ABHR containers to HCWs, and hand hygiene training sessions were organized in the previous year.

Our results suggest that the problem of low compliance with hand hygiene is very significant in Hungarian LTCFs. Several studies demonstrated reduction in HAI rates following hand hygiene promotion interventions; in particular, pneumonia and skin and soft tissue infections declined from 0.97 and 0.30 to 0.53 and 0.25 infections per 1000 resident days, respectively, in LTCFs in Taiwan and Hong Kong [17, 27]. Therefore, there is a need for multimodal, multidisciplinary hand hygiene promotion strategies, including staff education, performance monitoring and feedback, and the introduction or maintenance of point of care ABHR in Hungarian LTCFs.

Despite the fact that 94.1 % of Hungarian LTCFs conducted hand hygiene education in the previous year, our surrogate estimates of compliance proved very low in the current study. We presume that in-service education was based on insufficient knowledge of local trainers. Thus, developing and implementing a multimodal national education program by experts based on the WHO Guidelines on Hand Hygiene in Health Care would constitute a critical element for improvement in these settings [18].

Although the availability of ABHR alone is not sufficient to improve hand hygiene practices, several studies have demonstrated the positive influence of the use of pocket-sized containers and point of care ABHR on compliance with hygienic handrubbing [13, 28, 29]. The use of pocket-sized ABHR containers as a part of a multifaceted hand hygiene promotion strategy was effective to increase adherence and decrease HAIs incidence rates by 40–45 % in LTCFs with an elderly population [30–34].

While bacterial infections are still amongst the most frequent causes of morbidity and death in LTCFs, the prevalence of multidrug resistant organisms as causative agents of these infections is increasing worldwide. In fact, LCTFs are now considered a major reservoir of these microorganisms from where they can spread to both acute-care facilities and the community. In this scenario, hand hygiene promotion is essential for preventing the emergence of antimicrobial resistance and controlling its spread within and outside LTCFs [35, 36].

Our study has limitations. First, confidence on self-reporting restrained the objectivity of the collected data. Second, monitoring of ABHR consumption provides only an estimate of the possible number of hand hygiene actions; estimates are based on recommended volumes of agents used and not on real use. Furthermore, ABHR could have been used by residents or visitors on some occasions and the study did not capture it. Third, considering that the surveyed Hungarian facilities were not part of a random sample, it remains not clear whether participating LTCFs (24 %) are representative of the whole reference population. Finally, international comparisons are not feasible because no research has indirectly measured the number of hand hygiene actions among HCWs in LTCFs.

Based on the results of the current study, the National Center for Epidemiology (NCE) at the Department of Hospital Epidemiology and Hygiene will start a module for ABHR consumption as a long-term monitoring tool from 2015. Reporting will be on a voluntary basis with the aim of providing benchmarks with defined stratifications to allow for the comparison of results between similar LTCFs. This module will be integrated as an additional component into the existing web-based National Nososcomial Surveillance System (Nemzeti Nosocomiális Surveillance Rendszer, NNSR). Participating LTCFs will be informed yearly about their individual results with reference data and will be able to use their data for feedback and improvement in their institutions.

## Conclusions

This study provides one of the first large scale summary of the consumption of hygienic handrub and handwashing soap in LTCFs. Our findings suggest that there is an urgent need for educational interventions to improve hand hygiene practices in Hungary, not only by LTCFs directors and HCWs at local level, but also by stakeholders or lawmakers at national level. In addition, our study provides a foundation for further work to explore other factors (e.g., barriers to hand hygiene) which efforts to improve and promote proper hand hygiene practices in LTCFs.
